# Analysing incompliant attitudes towards antibiotic prescription completion in the UK

**DOI:** 10.1093/jac/dkz492

**Published:** 2019-12-04

**Authors:** Alistair Anderson

**Affiliations:** School of Geographical Sciences, University of Bristol, University Road, Bristol BS8 1SS, UK

## Abstract

**Objectives:**

To analyse demographic, social and geographic predictors of incompliant attitudes towards prescription completion in the UK.

**Methods:**

Two waves of the Eurobarometer survey (85.1 and 90.1) were analysed, with a final sample size of 2016. Using logistic regression, the best-fitting combination of a set of identified variables was specified. The regression output and the model-averaged importance of each variable were analysed.

**Results:**

Compared with a median prevalence region, respondents in the Nomenclature of Territorial Units for Statistics (NUTS) 1 London (OR = 2.358, 95% CI = 1.100–5.398) and Scotland (OR = 2.418, 95% CI = 1.083–5.693) regions were most likely to report an incompliant attitude. Respondents who correctly answered questions about whether unnecessary use of antibiotics could make them ineffective in future (OR = 0.353, 95% CI = 0.230–0.544), whether antibiotics kill viruses (OR = 0.644, 95% CI = 0.450–0.919) and whether antibiotics treat colds (OR = 0.412, 95% CI = 0.287–0.591) were less likely to report incompliant attitudes. Conversely, respondents who correctly responded that antibiotics can cause side effects (OR = 1.419, 95% CI = 1.014–1.999) were more likely to report incompliant attitudes. There was some evidence of associations between political orientation and level of compliance. Uncooperative survey respondents (OR = 2.001, 95% CI = 1.108–3.526) were more likely to report incompliant attitudes.

**Conclusions:**

Incompliant attitudes towards antibiotic prescription compliance in the UK are associated with a variety of factors, including regional geographic variation in attitudes. Knowledge about antibiotics can relate to good stewardship attitudes, but concerns over side effects are associated with poor attitudes. Further research should examine the underlying attitudes and beliefs that political orientation may be a marker for in the context of antibiotic stewardship. Survey samples reliant on self-selection are likely to be biased towards good stewardship.

## Introduction

Antimicrobial resistance (AMR) has a biological mechanism and socially patterned drivers and consequences.^1^^,^^2^ One example of such a driver is incompliance with prescription instructions leading to patients underdosing and potentially later self-medicating with antibiotics. The NHS website^3^ informs the public that ‘taking antibiotics when you do not need them can mean they will not work for you in the future’ and highlights that antibiotics should be taken ‘as instructed by your GP or pharmacist’. Compliance with instructions for medication-taking is influenced by several identified factors, including age, patient–physician relationship, beliefs about medications, misconceptions about disease conditions, experience and management of side effects and individual personality traits.^4–6^ Meta-analyses of adherence to medication have shown that individuals believing that the medication is necessary for their health are more likely to follow medication-taking instructions, while individuals who have strong concerns about the medication such as beliefs about side effects are less likely to follow instructions.^4^^,^^7^ In the Wellcome Monitor survey, 60% of respondents who reported not taking their antibiotics as prescribed said it was because they felt better, with 25% saying that it was because they experienced side effects.^8^

One mechanism of action in AMR public health interventions is the raising of public awareness through education.^9^ This occurs through the provision of information about consequences of inappropriate antibiotic use alongside information about how to take antibiotics appropriately and, commonly, the use of credible professional sources for intervention implementation. Interventions have had mixed results when targeting the general public and it has been argued that in addition to improving understanding of appropriate antimicrobial use, interventions should promote the role of the public in addressing AMR and its risks for individuals, their loved ones and the wider population.^10^

Increased knowledge about antibiotics and AMR has been found to correlate inconsistently with behavioural outcomes. Knowledge about antibiotics and AMR has been associated with good stewardship attitudes and behaviours^11–17^ as well as with negative behaviours such as self-medication or possession of leftovers.^17^^,^^18^ Relationships between prior knowledge, attitudes and behaviour around antibiotics are key areas of interest for interventions aiming to improve antibiotic stewardship in the community and this interest is addressed in this study by examining associations between specific areas of prior knowledge and attitudes towards prescription compliance.

Political orientation has been suggested as a marker for underlying attitudes, values and beliefs relating to health.^19^ Individual-level political orientation has been correlated with health^19^^,^^20^ and health-related attitudes and behaviours,^21–24^ and research in political psychology has argued that left- and right-orientated individuals have substantively different thought styles, with liberal/leftist and conservative/rightist political ideologies being associated with differing psychological needs.^25^^,^^26^ Conservative ideology, for example, has been positively associated in meta-analyses with uncertainty avoidance and intolerance for ambiguity,^26^ traits that have also been associated with higher national levels of antibiotic consumption using Hofstede’s Uncertainty Avoidance national-level cultural dimension.^27^ Individuals with different political orientations may think about health issues differently with possibly different attitudinal or behavioural outcomes such as compliance with antibiotic prescription instructions. These differences may impact the effectiveness of public health interventions’ framings.

Surveys are widely used to examine attitudes and behaviours regarding antibiotic consumption. Faster and lower-cost non-probability sampling methods that are reliant on self-selection and lack specifiable probabilities of selection for each included observation are becoming increasingly popular^28^^,^^29^ and have been used in the study of antibiotic use.^13^^,^^16^^,^^30^ While respondents in a non-probability sample may be demographically identically distributed to a probability sample, they are not necessarily attitudinally or behaviourally identical.^31^ An important consideration for survey research is the behaviour of respondents, for example their motivation and willingness to provide good-quality data and whether participation itself is correlated with attitudinal or behavioural outcomes of interest. This study examined whether survey cooperation is an issue for the measurement of attitudes towards prescription compliance through the analysis of a random probability survey sample (in which non-response can be adjusted for) with a survey interviewer-recorded variable for respondent cooperation.

The primary aim of this study was to examine predictors of variation in the UK public’s attitude towards antibiotic prescription compliance. This study also examines the relative importance of chosen candidate variables for the prediction of incompliant attitudes among the general public.

## Materials and methods

### Data and analysis

The data for this study were drawn from Eurobarometers 85.1^32^ and 90.1.^33^ The Eurobarometer uses a stratified sampling approach in a random probability sampling methodology. The combined 2016 and 2018 UK samples contain 2330 observations. To compare models, rows with missing values on candidate variables were excluded so that each candidate model would be analysing identical samples. The final subset contained 2016 cases. Supplied non-response weights incorporating sex, age, Nomenclature of Territorial Units for Statistics (NUTS) 2 regions and size of locality were used in the analysis.^32^^,^^33^

### Variable and model specification

The dependent variable for logistic regression in this study was based on the question ‘When do you think you should stop taking antibiotics once you have begun a course of treatment?’. The response was binary coded with the base as ‘When you have taken all of the antibiotics as directed by your doctor’ and the contrast as ‘When you feel better’, ‘Other’ and ‘Don’t know’. The independent variable representing political orientation was measured in the survey by self-placement on a 10-point scale from left to right and condensed for this study to five categories (1–2 = left, 3–4 = centre-left, 5–6 = centre, 7–8 = centre-right, 9–10 = right).

Model and variable selection was undertaken using the package glmulti^34^ in RStudio. Candidate predictor variables were chosen from the Eurobarometer dataset and were fitted in all combinations of 20 main effects. The best 100 models based on lowness of Akaike Information Criterion (AIC) were stored and used to determine the best-fitting model (model with lowest AIC,^35^ AIC_min_) and relative importance of each candidate variable.

**Figure 1.**
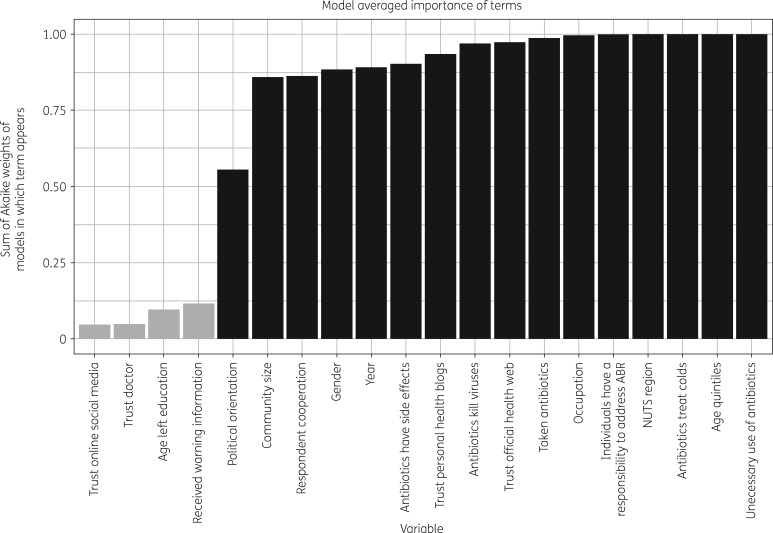
Model-averaged importance of candidate variables, with those included in the best-fitting (AIC_min_) regression model shaded black.

The relative importance of the 20 candidate variables is presented in Figure 1 in terms of the summed Akaike weights of models in which the variable appears. Each model’s Akaike weight is calculated as the relative likelihood of each model in the candidate set (for model *i*: $\text{exp}\;(-\frac{{\mathrm{AIC}}_{i}-{\mathrm{AIC}}_{\min}}{2})$) divided by the summed relative likelihoods of all 100 candidate models, representing the probability that model *i* is the best model for the data in the set of specified models.^35^

Regional and community geographies were consistently important for examining variation in attitudes towards prescription compliance, along with certain demographic characteristics and respondents’ cooperation with their survey interviewer. Antibiotic-related variables of importance were specific areas of knowledge about antibiotics and antibiotic resistance (ABR), respondents’ perception of whether individuals have a role in addressing ABR and reporting trust in official health websites and personal blogs as sources of information about antibiotics. Time spent in education, trust in either doctors or social media for information about antibiotics, the presence of children in the household and recent reception of warning information about not taking antibiotics unnecessarily were rarely present in the best 100 models and consequently may be considered less important for explaining variation in compliance attitudes. The model with AIC_min_ was considered the best-fitting model and included 16 variables, which are shaded black in Figure 1.

## Results

Of 2016 respondents, 220 (11% of the sample) reported an attitude response other than taking antibiotics as directed by their doctor. The results of the multivariable AIC_min_ logistic regression model are presented in Table 1 with estimated ORs and 95% confidence limits (CLs). Statistical significance was determined using CLs, with ORs where the interval between CLs [the confidence interval (CI)] did not include one considered significant at a 95% level of confidence.

**Table 1.** Results of best-fitting (AIC_min_) multivariable regression model

Independent variables	OR	2.5% CL	97.5% CL
Age (years)			
15–28	(reference)	(reference)	(reference)
29–40	0.642^a^	0.413	0.995
41–52	0.493^a^	0.303	0.794
53–66	0.257^a^	0.144	0.444
67+	0.221^a^	0.121	0.391
Community size			
large urban	(reference)	(reference)	(reference)
small urban	0.626^a^	0.417	0.945
rural	0.739	0.380	1.386
Employment			
not working	(reference)	(reference)	(reference)
self-employed	2.034^a^	1.160	3.519
employed	0.755	0.518	1.100
Level of cooperation			
excellent	(reference)	(reference)	(reference)
fair	1.326	0.838	2.059
average/bad	2.001^a^	1.108	3.526
Region			
East Midlands	(reference)	(reference)	(reference)
London	2.358^a^	1.100	5.398
East of England	0.643	0.193	1.918
North East England	0.151^a^	0.010	0.818
North West England	2.130	0.947	5.033
Northern Ireland	1.018	0.450	2.415
Scotland	2.418^a^	1.083	5.693
South East England	1.357	0.604	3.196
South West England	1.099	0.402	2.941
Wales	1.452	0.447	4.327
West Midlands	1.024	0.430	2.509
Yorkshire and The Humber	1.779	0.767	4.301
Political orientation			
centre	(reference)	(reference)	(reference)
left	1.797^a^	1.010	3.115
centre-left	0.646^a^	0.409	0.998
centre-right	0.900	0.529	1.485
right	0.712	0.271	1.642
don’t know or refuse	0.577	0.257	1.209
Sex			
female	(reference)	(reference)	(reference)
male	1.479^a^	1.064	2.067
Antibiotics taken in past 12 months on prescription			
no	(reference)	(reference)	(reference)
yes	0.692	0.470	1.004
Year			
2016	(reference)	(reference)	(reference)
2018	0.863	0.723	1.028
Trust in information sources			
trust in source not mentioned	(reference)	(reference)	(reference)
trust in official health web for information mentioned	0.571^a^	0.332	0.941
trust in personal health blog for information mentioned	0.160	0.003	1.203
Antibiotic knowledge			
incorrect response to each question	(reference)	(reference)	(reference)
antibiotics kill viruses	0.644^a^	0.450	0.919
antibiotics can treat colds	0.412^a^	0.287	0.591
unnecessary use of antibiotics can make them ineffective	0.353^a^	0.230	0.544
antibiotics commonly cause side effects	1.419^a^	1.014	1.999
Level at which ABR should be addressed			
level other than individual	(reference)	(reference)	(reference)
individual or family level	1.839^a^	1.294	2.599

a Denotes evidence of significance at a 95% level of confidence.

### Demographics

Incompliant attitudes towards doctors’ instructions regarding antibiotics were associated with multiple demographic characteristics. Older members of the public were less likely to report an incompliant attitude towards doctors’ instructions (29–40 years OR = 0.642, 95% CI = 0.413–0.995; 41–52 years OR = 0.493, 95% CI = 0.303–0.794; 53–66 years OR = 0.257, 95% CI = 0.144–0.444; 67+ years OR = 0.221, 95% CI = 0.121–0.391). These results suggest that levels of compliance with antibiotic prescription instructions are higher among older members of the public and that this association is clearer in the oldest quintiles compared with younger quintiles. Male (OR = 1.479, 95% CI = 1.064–2.067) respondents were more likely to report an incompliant attitude than female respondents, whilst self-employed respondents (OR = 2.034, 95% CI = 1.160–3.519) were more than twice as likely to report an incompliant attitude than respondents who were not in work. Respondents who had been prescribed antibiotics in the past 12 months (OR = 0.692, 95% CI = 0.470–1.004) were not substantially different from respondents who had not. Alongside this, recent reception of warning information was a relatively unimportant variable in the model selection process, which suggests that recency of contact with either a healthcare professional or intervention are substantially less important for explaining variation in compliance than other candidate variables included in this analysis.

### Respondent survey cooperation

Respondents who were categorized as having average or bad cooperation during the survey interview (OR = 2.001, 95% CI = 1.108–3.526) were twice as likely to report an incompliant attitude towards antibiotic prescription instructions than excellent cooperators. This suggests that members of the public who are less motivated to take part in surveys and provide good-quality data are more likely to be individuals who exhibit poorer attitudes towards antibiotic stewardship, independent of other factors such as knowledge or age.

### Geography

**Figure 2.**
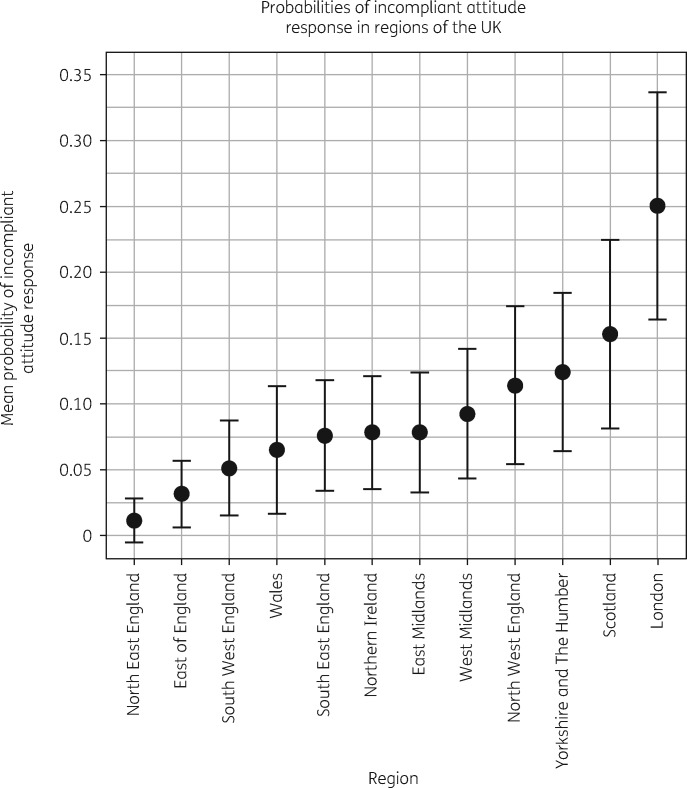
Mean predicted probabilities of incompliant attitude responses in each region of the UK. CIs calculated using the Goldstein and Healy^37^ procedure for graphical comparison of multiple means.

There is regional variation in the predicted probability of respondents reporting incompliant attitudes, shown in Figure 2. The smallest region size available in these data is NUTS 1 level, with populations between 3 and 7 million people.^36^ Respondents in North East England had the lowest and most precise probability of reporting an incompliant attitude and the probabilities associated with the East of England and South West England were also both relatively low and precise. Compared with the differences between most regions’ means, which were relatively small, Londoners were substantially more likely to report an incompliant attitude despite a wide CI.

When contrasted with the East Midlands (one of the median regions in terms of proportion of incompliant responses) there were three significantly different areas in the regression. Respondents in London (OR = 2.358, 95% CI = 1.100–5.398) and Scotland (OR = 2.418, 95% CI = 1.083–5.693) presented higher likelihoods of incompliant responses. Conversely, respondents in North East England (OR = 0.151, 95% CI = 0.010–0.818) were less likely to report an incompliant response.

**Figure 3.**
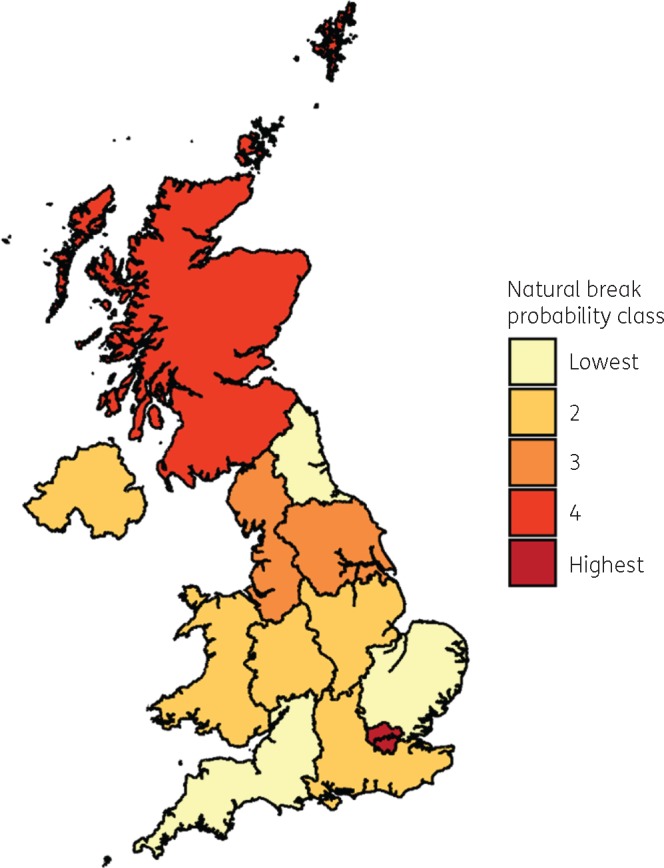
Map of regional mean predicted probabilities of incompliant attitude responses, grouped by natural breaks detailed in Tables S1 and S2 (available as Supplementary data at *JAC* Online). This figure appears in colour in the online version of *JAC* and in black and white in the print version of *JAC*.

These results suggest that a regional geography at the NUTS 1 level, visualized using predicted probabilities in Figure 3, persists after controlling for other factors including local geography.

The regression suggests that the local geography of incompliant attitudes is predominantly urban. Respondents who lived in small urban areas (OR = 0.626, 95% CI = 0.417–0.945) were less likely than respondents in large urban areas to respond that they would not adhere to a doctor’s instructions when taking antibiotics and there was no significant association for rural (OR = 0.739, 95% CI = 0.380–1.386) respondents contrasted with respondents from large urban areas.

### Knowledge about antibiotics

Four knowledge questions were included in the model. Correct responses to three of these questions were associated with lower likelihoods of respondents reporting incompliant attitudes. The strongest of these associations was for whether unnecessary use of antibiotics could make them ineffective in future (OR = 0.353, 95% CI = 0.230–0.544), followed by whether antibiotics are useful to treat colds (OR = 0.412, 95% CI = 0.287–0.591) and whether antibiotics kill viruses (OR = 0.644, 95% CI = 0.450–0.919). These results suggest that the piece of knowledge most strongly associated with a higher likelihood of prescription compliance is knowledge about the relationship between antibiotic overuse and ABR. Conversely, respondents who correctly answered that antibiotics commonly cause side effects (OR = 1.419, 95% CI = 1.014–1.999) were more likely to report incompliant attitudes.

### Attitudes: trusted information, political orientation and individual-level roles

Of the two trusted information sources included in the model, only trust in official health websites for information about antibiotics (OR = 0.571, 95% CI = 0.332–0.941) was significantly associated with compliant attitudes. This could mean that the official health websites in the UK are successfully communicating that antibiotic prescriptions should be finished. However, it could also mean that respondents who are more inclined to trust government-sourced online health information are also respondents who are more likely to be compliant with their doctor’s instructions anyway. In either case, this result suggests that there are substantially different groups of internet users in the context of behaviours regarding antibiotic prescriptions. Very few (*n *=* *18) respondents reported trusting personal health blogs, which likely explains the wide CIs.

Left orientation had a stronger association with prescription compliance than right orientation. Respondents who placed themselves on the left of the scale (OR = 1.797, 95% CI = 1.010–3.115) compared with in the centre were more likely to respond that they would not adhere to a doctor’s instructions when taking antibiotics. In contrast, centre-left-placed respondents (OR = 0.646, 95% CI = 0.409–0.998) were less likely to report an incompliant attitude. The results suggest a greater association between left placement than right placement and attitudes towards prescription compliance; however, the pattern of this relationship is not a clear image of left-leaning individuals in general having specific predilections towards or against compliance.

The regression suggests that perceptions of personal responsibility matter for prescription compliance. Independently of political orientation, respondents who believed that it is ‘most effective to tackle the resistance to antibiotics’ at the individual level (OR = 1.839, 95% CI = 1.294–2.599) as opposed to regional, national, EU or global levels were more likely to report an incompliant attitude. These results suggest perceptions of personal responsibility in addressing ABR are associated with prescription compliance independent of political orientation, which itself could be a marker for compliance-related attitudes among groups of left-leaning individuals.

## Discussion

Incompliance with prescription instructions leading to patients underdosing and potentially later self-medicating with antibiotics is a socially patterned driver of AMR. There is debate over the validity of generic advice to complete courses of antibiotics;^38–41^ however, the prevailing advice from the NHS is to consume antibiotics as directed by a healthcare professional.^3^ A set of variables in the Eurobarometer surveys was used in this study to examine variation in attitudes to antibiotic prescription compliance in the UK.

### Implications of geographic findings

The findings of this study suggest that there is geographic variation in compliance attitudes regarding antibiotic prescriptions in the UK that persists independently from several individual-level factors. This study suggests that respondents in small urban areas are less likely to report an incompliant attitude towards following a doctor’s instructions when taking antibiotics. There is also evidence of a regional geography persisting once several individual-level factors are accounted for. In terms of the regions analysed in this study, the evidence from regression suggests that this geographical variation manifests at the extremes, with most regions not significantly different from the median. Respondents in London and Scotland, for example, are more likely to report incompliant attitudes than median region respondents, while respondents in North East England are less likely to do so. A limitation of this analysis is the resolution of the regions available for analysis.

Further research should examine this geography of attitudes at a higher resolution to enable a clearer comparison with geographies of prescribing such as those presented by Curtis *et al*.,^42^ for example on dimensions of deprivation, population or cultural characteristics. If high-prescribing areas are positively correlated with areas exhibiting higher levels of poor attitudes to prescription compliance, this could suggest prioritization of specific areas requiring attention from public health interventions to improve prescription practice and compliance by patients. These geographies may, however, have different characteristics, as Curtis *et al*.^42^ found that ruralness was associated with higher levels of prescribing, whilst this study suggests that rural areas are not significantly different from large urban areas in terms of attitude. Instead, attitudinal differences in prescription compliance manifest between large and small urban areas.

### Implications of knowledge-related findings

This study provides further evidence of an association between specific areas of respondents’ knowledge about appropriate use of antibiotics and ABR, and attitudes towards antibiotic prescription compliance. Whilst the data are cross-sectional and limiting to causal inference, this analysis suggests that members of the public who are aware that antibiotics are not effective against colds and other viral infections, and that unnecessary use of antibiotics can lead to them becoming ineffective in future, are less likely to be incompliant with prescriptions. This may reflect the commonness suggested by McParland *et al*.^9^ of information about the consequences of inappropriate use alongside information on how to take antibiotics appropriately in AMR public health interventions. Conversely, and in line with findings from meta-analyses on necessity/concerns framework beliefs relating to medication,^4^^,^^7^ respondents who correctly responded that antibiotics cause side effects were more likely to report an incompliant attitude towards finishing their prescription. The most desirable message for public health interventions suggested by this study is that unnecessary use of antibiotics can render them ineffective in future.

Respondents who reported trust in official health websites for information about antibiotics were less likely to hold an incompliant attitude towards antibiotic prescriptions. Neither trust in doctors nor social media were important predictors of compliance attitudes. This is a more specific finding than that which has been previously reported from analysis of this data.^11^ These findings may suggest that information dissemination through the official websites, such as that of the NHS, has had a positive impact on antibiotic stewardship; however, it may also mean that members of the public who are already likely to comply with a doctor’s instructions are also more trusting of ‘credible’ professional online sources of information through which interventions are implemented.

### Political orientation in context of other research

Political orientation has been suggested as a marker for underlying health-related beliefs and attitudes.^19^ Whilst movement towards right orientation has been associated with more medicalized attitudes and other health-related behaviours in previous studies,^21–24^ there was no evidence of substantial difference found in this study between respondents who placed themselves on the political right in contrast to the centre in terms of compliant attitudes. There were, however, differing associations between left-leaning placements and the centre in terms of antibiotic prescription compliance, as incompliance was more likely for left-placed respondents and less likely for centre-left-placed respondents. Independently, respondents who believed the individual level was the most effective level at which to address ABR were more likely to be incompliant than those who believed the most effective level was above the individual.

Social politics has been proposed as a better predictor of thought than economic politics,^25^ but without the data to examine social and economic politics separately the inferences that can be made from the associations in this study are limited. For example, the lack of evidence for association between right-leaning orientations and prescription compliance could be because there is no association between right placement and prescription compliance, but it could also be due to bias from differences between libertarians and social conservatives within this wing of the scale.^25^ Similarly, left- and centre-left-placed respondents were differently associated with compliance, suggesting that in terms of this health-related attitude, there is some substantive difference between groups that cannot be illuminated further with this data. This difference could, for example, relate to contextually bound obedience to authority^43^ (centre-left respondents may perceive doctors as being on their social or political ‘team’, for example), differences in underlying psychological needs,^26^ or styles of thought or morality^25^ (for example, left-placed respondents may be averse to institutional authority in the context of health).

Further research should examine the relationship between differently politically orientated antibiotic consumers and their levels of compliance, with attention to cognitive styles and social/economic politics, as this may be suggestive of specific and effective framings for future public health interventions addressed to the different thought styles of these groups.

### Implications of survey interview-related findings

The positive association presented in the model for respondents who were reported by interviewers as having had average or bad levels of cooperation has implications for future survey research in this area. The deployment of non-probability sampling approaches, which have been used in the area of antibiotic use,^13^^,^^16^^,^^30^ relies on the self-selection of respondents into surveys, which can lead to biases on attitudinal and behavioural measures even where samples are demographically representative. These biases are introduced because individuals who self-select for specific studies are different on both measured and unmeasured characteristics, such as agreeableness and interest in the topic, than individuals who do not take part. Random probability samples such as those used in this study do not exhibit these biases because unmeasured characteristics in the wider population are randomly sampled along with the measured variables. The findings of this study suggest that members of the public who are less motivated to take part in surveys and provide good-quality data are also individuals who are more likely to exhibit poorer attitudes towards antibiotic stewardship. This means that non-probability-based inferences are likely to be biased towards respondents with better stewardship attitudes and that greater efforts will need to be expended to reach incompliant individuals and avoid samples based predominantly on agreeable and interested respondents.

### Conclusions

Incompliant attitudes towards antibiotic prescription compliance in the UK are associated with a variety of factors including local and regional geography, prior knowledge about antibiotics and ABR, and demographics characteristics. There may be an association between political orientation as a marker for underlying attitudes and antibiotic prescription compliance and more specific research is needed to examine this area. Finally, survey respondents who are less motivated to take part in surveys are also more likely to report incompliant attitudes towards antibiotic prescriptions. This suggests that biases in survey data from samples reliant on self-selection may be a significant problem for the measurement of prescription incompliance attitudes.

## Supplementary Material

dkz492_Supplementary_Data
